# Anterior Inferior Cerebellar Artery Aneurysm Mimicking a Vestibular Schwannoma

**DOI:** 10.7759/cureus.21807

**Published:** 2022-02-01

**Authors:** Tania Benjamin, Nicole T Jiam, Daniel Cooke, Michael C Huang, Jeffrey D Sharon

**Affiliations:** 1 Otolaryngology, University of California San Francisco, San Francisco, USA; 2 Radiology and Biomedical Imaging, University of California San Francisco, San Francisco, USA; 3 Neurosurgery, University of California San Francisco, San Francisco, USA

**Keywords:** aneurysm, hearing loss, vertigo, vestibular schwannoma, aica

## Abstract

Anterior inferior cerebellar artery (AICA) aneurysms are rare pathologies that may present with hearing loss, facial paralysis, vertigo, and tinnitus. Otologic symptoms at the time of presentation may prompt physicians to order an MRI, which can lead to the misdiagnosis of AICA aneurysms as vestibular schwannomas. We discuss the case of a 27-year-old female who presented with sudden-onset vertigo and right-sided hearing loss. She was found to have a right homogeneously enhancing internal auditory canal (IAC) mass abutting the vestibular nerve on post-gadolinium T1 MRI two hours after the presentation, which was initially diagnosed as a vestibular schwannoma. Serial T1 MRI highlighted the evolution of blood products within this mass by presenting as bright at two days and dark at two months after presentation. Profound ipsilateral sensorineural hearing loss and absent vestibulocochlear function were confirmed on audiometry and vestibular testing, respectively. The diagnostic cerebral angiogram was complicated by an iatrogenic right mid-cervical vertebral artery dissection, and the patient ultimately underwent successful embolization two months after presentation with the resolution of all presenting symptoms except right-sided hearing loss. Early recognition and treatment of an AICA aneurysm may help prevent associated vascular complications, and they should be considered as part of the differential diagnosis for IAC lesions despite their rarity.

## Introduction

Aneurysms of the anterior inferior cerebellar artery (AICA) are rare entities that comprise less than 1% of all intracranial aneurysms and are challenging to manage in general. Symptoms associated with AICA aneurysms reflect its close proximity to the internal auditory canal (IAC) and include hearing loss, facial paralysis, vertigo, and tinnitus. While they can be readily diagnosed with a CT angiogram or digital subtraction angiography (DSA), an MRI may be the first imaging modality obtained when patients present with otologic symptoms [1]. The differential diagnosis for IAC masses also includes vestibular schwannomas, lipomas, and meningiomas. Although signs to differentiate between the two on MRI have been published, AICA aneurysms continue to be mistaken as vestibular schwannomas given their rarity [2]. This case report outlines the importance of considering AICA aneurysms as part of the differential diagnosis for IAC lesions with unusual MRI findings.

## Case presentation

**Figure 1:**
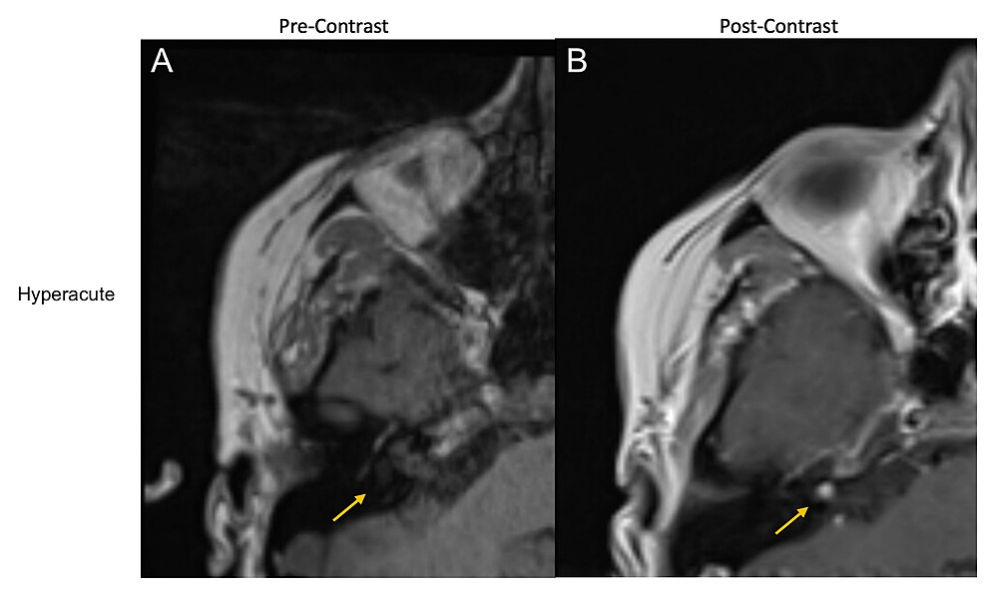
Right IAC lesion on MRI with and without contrast at hyperacute (time of presentation) time point A: Pre-gadolinium T1-weighted axial MRI brain demonstrating the right IAC during hyperacute presentation with the isointense appearance of the lesion B: Post-gadolinium T1-weighted axial MRI brain showing a rounded 4-mm homogenously enhancing lesion in the right IAC (hyperacute) IAC: internal auditory canal; MRI: magnetic resonance imaging

A 27-year-old healthy female presented to the emergency department with sudden-onset right-sided hearing loss and vertigo. On exam, she was noted to have left-beating nystagmus in all directions, a wide-based and unsteady gait, and intact facial movement. A code stroke was activated. A CT angiogram was negative for intracranial hemorrhage or ischemia, including no AICA or posterior inferior cerebellar artery (PICA) occlusions. MRI of the head two hours later showed a homogeneously enhancing right IAC mass (~4 mm) abutting the vestibular nerve without associated reduced diffusion, which was interpreted as a small vestibular schwannoma (Figures 1A, 1B).

**Figure 2:**
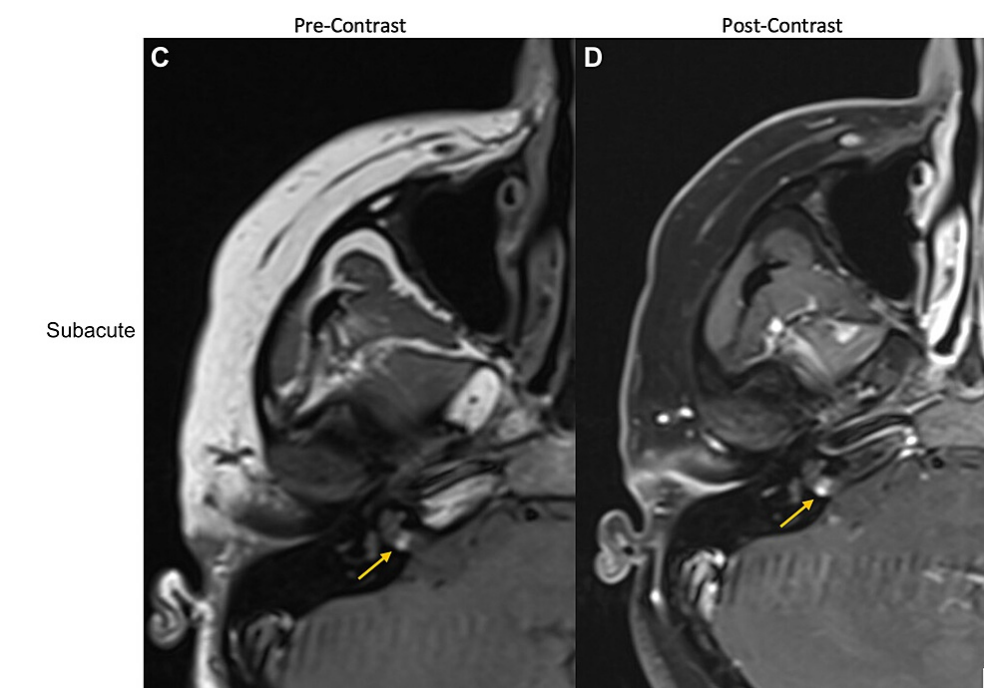
Right IAC lesion on MRI with and without contrast at subacute (two days after presentation) time point C: Pre-gadolinium T1-weighted axial MRI IAC during early subacute period showing a hyperintense right IAC lesion D: Post-gadolinium T1-weighted axial MRI IAC showing a homogeneously enhancing 3 x 5-mm soft tissue lesion in the right IAC IAC: internal auditory canal; MRI: magnetic resonance imaging

A dedicated MRI of the IAC nearly two days after the initial presentation confirmed these findings (Figures 2C, 2D), with a small change noted in hindsight: the lesion was now intrinsically bright on T1.

The patient was discharged with meclizine and lorazepam (advised to be taken as needed) for symptom control along with a scheduled follow-up with otolaryngology and neurosurgery for surgical evaluation.

**Figure 3:**
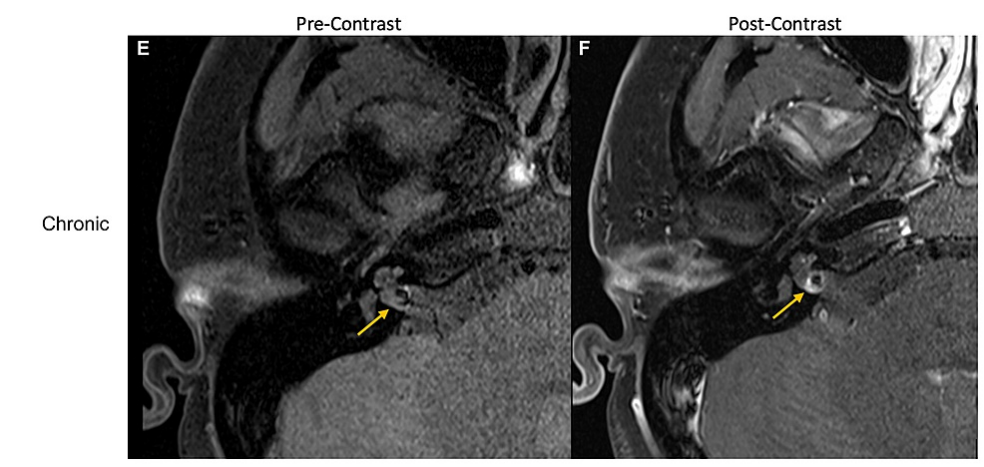
Right IAC lesion on MRI with and without contrast at chronic (two months after presentation) time point E: Pre-gadolinium T1-weighted axial MRI IAC showing an isointense IAC lesion with central hypointensity two months after the initial presentation F: Post-gadolinium T1-weighted axial MRI IAC showing the lesion as T1 hypointense with T1 intrinsic hyperintensity and with a surrounding margin of enhancement on postcontrast imaging, consistent with a chronic hemorrhage. These images follow the expected evolution of T1 precontrast appearance for blood products after an acute hemorrhage IAC: internal auditory canal; MRI: magnetic resonance imaging

In the subsequent weeks, audiometry revealed profound right-sided sensorineural hearing loss across all frequencies, a 0% score on word recognition testing, and absent ipsilateral acoustic reflexes. Vestibular testing showed absent ocular evoked myogenic potential (oVEMP) and cervical VEMP (cVEMP), and video head impulse test (vHIT) was notable for low gain with overt and covert saccades in all three right semi-circular canals, diagnostic of right peripheral vestibular weakness. Based on disabling vertigo and absent vestibulocochlear function, the patient elected to undergo a right translabyrinthine approach for the removal of the IAC lesion. However, on repeat preoperative MRI of the IAC two months after the initial presentation, the right IAC lesion was noted to have changed. It was now heterogeneous, with a central hypointense area, and a peripheral rim that enhanced with contrast (Figures 3E, 3F).

**Figure 4:**
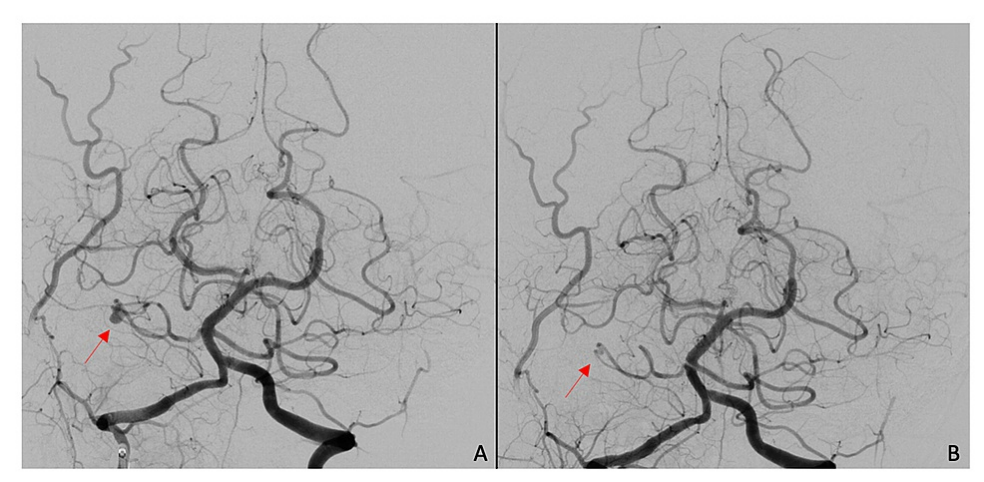
CT angiogram (A) pre-embolization and (B) post-embolization, demonstrating the absence of filling of right AICA AICA: anterior inferior cerebellar artery; CT: computed tomography

Notably, the central area of the low signal appeared to correspond to a site of arterial enhancement on a prior CT angiogram. The signal and enhancement characteristics appeared to be more consistent with a vascular malformation or aneurysm. A diagnostic cerebral angiogram showed a 6 x 3 x 3-mm fusiform right AICA aneurysm of the lateral pontine segment (Figure 4A), which was complicated by an iatrogenic right mid-cervical vertebral artery dissection. Two months later, the patient underwent a successful endovascular embolization of the AICA aneurysm and parent vessel sacrifice using 30% n-butyl cyanoacrylate (NBCA) (Figure 4B). At three months post-embolization, the patient’s vertigo, gait imbalance, and nystagmus had resolved, but her right-sided hearing loss remained unchanged from pre-embolization. She had not had a post-embolization audiogram.

## Discussion

AICA aneurysms may be misdiagnosed as vestibular schwannomas depending on the initial imaging obtained, but they are more readily identifiable on CT angiogram or DSA. Unusual MRI findings should raise the suspicion for an AICA aneurysm; in particular, the absence of IAC enlargement and a “blurry dot sign,” indicating blood flow artifacts seen with and without contrast, have previously been described as MRI findings consistent with AICA aneurysms [2]. In addition, this patient’s initial symptoms of sudden-onset and disabling vertigo are rare in vestibular schwannomas, which also led to an alternative diagnosis. Management of an AICA aneurysm varies based on the segment of AICA affected, and successful management with endovascular or open surgical clip ligations has been reported in the literature [3]. However, there is no superior treatment modality in reversing existing deficits [4]. Early diagnosis of an AICA aneurysm may prevent associated complications of cerebellopontine irritation and subarachnoid hemorrhage, and avoid vascular complications that can arise if the lesion is mistaken for vestibular schwannoma. In this case, based on imaging obtained at multiple time points, we believe that there was a sudden hemorrhage into the aneurysm at the time of the initial presentation, causing the presenting symptoms. Subsequent imaging neatly followed the expected evolution of T1 signal for blood products - hyperacute: isointense, subacute: bright, and chronic: dark [5].

## Conclusions

We reported the case of a 27-year-old female who presented to the emergency room with sudden-onset right-sided hearing loss and vertigo and was initially believed to have a right-sided small vestibular schwannoma based on an initial MRI. Subsequent MRI images were notable for the evolution of T1 signal intensity, with the two-month MRI and prior CT angiogram findings leading to a diagnosis of a right AICA aneurysm of the lateral pontine segment. She underwent successful endovascular embolization, with a resolution of all symptoms except right-sided hearing loss that remained stable.

AICA aneurysms are rare pathologies but may be mistaken for vestibular schwannomas early in the presentation. The proximity of the aneurysms to the IAC leads to similar symptomatology, and in this case, vestibular testing confirmed absent vestibulocochlear function. Differentiation between AICA aneurysms and other masses depends on the detection of the evolution of blood products, or signal intensity, on serial head imaging.
